# India–Africa pharmaceutical cooperation and the future of global health

**DOI:** 10.1080/16549716.2026.2759419

**Published:** 2026-11-09

**Authors:** Dan Banik, Lise Bjerke

**Affiliations:** Centre for Global Sustainability, University of Oslo, Oslo, Norway

**Keywords:** Access to medicines, South-South cooperation, pharmaceutical manufacturing, India, Africa

## Abstract

As many Western donors retreat from the aid world and the multilateral health system fragments, the supply of essential medicines to the African continent increasingly depends on a production chain linking Chinese active ingredients, Indian medicines, and African distribution. We argue that the resilience and expansion of this South–South pharmaceutical arrangement, rather than the reconstruction of the old global health and aid architecture, will shape much of the African continent’s access to medicines in a multipolar and fragmented world order. In this scenario, we propose three recommendations for strengthening India–Africa pharmaceutical cooperation. First, to improve access to medicines and markets, there is a need to move from regulatory sovereignty to reliance models. Second, escaping the local manufacturing trap will require plants to serve markets beyond national borders, and intellectual property flexibilities represent an underutilized avenue to build manufacturing. Finally, durable India–Africa health cooperation will require replacing the paradigm of knowledge transfer and benevolent assistance with co-ownership and two-way learning.

## Paper Context

- **Main findings**: Many African countries’ access to medicines rests substantially on a South–South manufacturing and distribution chain, linking China, India and Africa, yet this configuration remains largely overlooked in current global health policy debates.
- **Added knowledge**: Recognising this South–South pharmaceutical arrangement is critical for advancing health security and sovereignty agendas on the African continent.
- **Global health impact for policy and action**: We propose three recommendations to strengthen India–Africa pharmaceutical cooperation, through regulatory reliance, local manufacturing, and two-way learning.

## Background

The established global health architecture is under pressure. In 2025, the United States withdrew from the World Health Organization (WHO) and Gavi, and in 2026, most donor countries from the Global North have failed to meet long-standing aid commitments. These developments have exposed how dependent many African health systems remain on Global North financing. African governments and institutions are now redirecting budgets, rebuilding manufacturing, and rethinking partnerships [1,2]. However, what has received less attention in recent academic and policy discourse is that the access to generic medicines in many African countries depended in part on a South–South production chain in which active pharmaceutical ingredients (APIs) made in China are formulated into finished medicines in India and distributed across African markets. An analysis of the future of global health must account for this arrangement, including its vulnerabilities and the conditions under which it can be strengthened.

This article builds on insights from qualitative interviews and roundtables with 20 Indian pharmaceutical industry leaders and regulators conducted between 2022 and 2025 in Hyderabad, Mumbai, and New Delhi, and interviews with more than 30 policymakers, regulators, practitioners, pharmacists and pharmaceutical company representatives in Malawi, South Africa, and Kenya. Participants were recruited through our networks and snowball sampling. To inform this article, roundtable and interview transcripts and notes were analysed thematically to identify recurring perspectives on India–Africa pharmaceutical cooperation.

The argument we present is three-fold. First, the resilience of medicine supply to many African countries depends not on India decoupling from China but on the two countries maintaining a stable and mutually dependent supply relationship. Second, the decline in global health financing from the Global North threatens the procurement mechanism that has supported some of that supply. Third, the most consequential reforms lie within Africa’s own regulatory and industrial architecture and in reshaping the India–Africa relationship from one based on assistance to one based on co-ownership.

## Health diplomacy by production

India’s engagement with the African continent differs from both Western aid and Chinese infrastructure finance. Its appeal is based substantially on the reputation of its pharmaceutical industry. India supplies over 50% of the continent’s demand for generics and is also an important source for pharmaceutical raw materials [3,4]. Indian drug companies are present in many African countries through joint ventures, acquisitions, or contract manufacturing for local firms [5]. India’s drug exports to Africa are channelled both through private commercial trade and through the procurement of national institutions, international organisations and global health partnerships. Indian manufacturers account for 70% of the global supply of antiretrovirals, 60% of UNICEF’s vaccine supplies, and 40 to 90% of WHO’s demand for DPT, BCG and measles vaccines [4].

The COVID-19 pandemic extended India’s health diplomacy vis-à-vis the continent. India and South Africa together proposed a temporary patents waiver to boost the supply of vaccines in the Global South [6]. The *Vaccine Maitri* initiative delivered Indian-manufactured vaccines to 42 African states [7]. Moreover, India’s sponsorship of permanent African Union membership in the G20 consolidated its claim to Global South leadership [8]. In normal times, India’s health diplomacy relies less on donations and more on supply through production of ‘Triple A’ technology: affordable, appropriate, and adaptable [9]. This diplomatic approach offers one response to the question of how countries can reduce their dependency on aid from the Global North while accelerating health leadership in low- and middle-income countries. Any answer to the dependency question remains incomplete unless it also examines the foundations currently underpinning pharmaceutical supply.

## The chemistry behind the pharmacy

If India is the ‘pharmacy of the world’ [10], China is the chemist behind the pharmacy. Approximately two-thirds of the APIs, key starting materials, and intermediates used by Indian manufacturers are sourced from China [11,12]. New Delhi’s dependence on Beijing is, however, not one-sided. Were India’s demand for active pharmaceutical ingredients to disappear, China alone would be unable to meet global pharmaceutical demand. Chinese ingredients are often further processed into final APIs on the Indian side [13]. India’s comparative advantage lies primarily in value-added APIs, formulations, and its capacity to meet diverse marketing authorisation requirements.

The implication for African health security is counterintuitive and politically inconvenient. It is the combination of Chinese chemistry and Indian formulation that delivers an affordable and audit-grade pill at all. Even African countries with substantial pharmaceutical manufacturing depend on APIs imported from India and China [5,14]. A key geopolitical determinant of African health is therefore the state of Sino-Indian and Indo-African relations. While acknowledging the importance of this South–South arrangement, a greater diversification of pharmaceutical supply chains through African manufacturing could further improve resilience and reduce vulnerability to geopolitical shocks.

## Access, procurement, and manufacturing in a post-aid era

Donor-financed procurement has improved access to certain medicines but has not addressed the corporate and technological concentration behind broader pharmaceutical market and supply failures [15]. In many African countries, medicine prices remain substantially higher as compared to the lowest international refence levels [16], and medicine costs are a major driver of out-of-pocket health spending [17]. In low-and-middle-income countries across the continent, essential medicines are frequently unavailable in government-run health facilities and priced beyond the reach of many patients at private clinics and pharmacies [18]. Managing supply is complex because medicines enter through multiple public, NGO, and private-sector supply chains. Countries also differ in their implementation of essential medicine lists, decentralised procurement, and manufacturing support [19]. African countries import between 70% and 100% of finished medicines, 99% of vaccines, and 90 to 100% of their API demand [20]. While India has nearly 10,500 manufacturing plants, there are 650 to 700 manufacturing plants in Africa, mainly manufacturing basic generics for local markets. These are heavily concentrated in a few countries, with South Africa, Egypt and Nigeria being home to roughly 60% of the plants [21,22].

The ongoing withdrawal of donor-funded procurement increases the risk of less stable access to certain medicines [23]. Aid withdrawal has already been followed by severe disruptions in access to HIV and TB medicines, as indicated by the experiences of South Africa, Uganda and Zimbabwe [24–26]. However, assuming that access to medicines will inevitably deteriorate without assistance from Western donors is a fallacy. Disruptions are actively responded to by African governments, seeking alternative and domestic sources of funding to increase local manufacturing [1]. Regional and international initiatives are emerging, including the African Pooled Procurement Mechanism [27], and an option to sustain a non-donor-funded pooled procurement through the established mechanisms of the Global Fund [28]. These instruments can help ensure low prices, quality, pricing transparency, reliable supply, and preference to African manufacturers. However, it requires stronger regulatory coordination, mutual trust, and guaranteed financing. Pooled procurement may also be less suitable where disease burdens, consumption patterns, or needs are particularly urgent, limited, or country-specific [29]. Multiple new procurement and supply models for a post-aid era are therefore urgently needed. It is in this context that the India–Africa pharmaceutical relationship assumes greater importance, but realising its potential will require changes in how the partnership is structured.

## Three reforms for a multipolar order

We propose three recommendations for strengthening India–Africa pharmaceutical cooperation.

### Regulatory reliance rather than sovereignty

Most African pharmaceutical markets are semi-regulated, as only 10 African national medicines regulatory authorities have attained WHO’s Maturity Level 3, the benchmark for a well-functioning regulatory system [30]. Differences in countries’ registration systems impose costs without commensurate protection, with countries demanding separate dossiers, fees, inspections, and timetables [31,32]. While approvals may take years, many of our informants claimed that national regulators often decline to treat a neighbouring country’s registration as sufficient. Explanations for this include limited institutional capacity, lack of trust, but also a financial incentive, as registration fees constitute a reliable revenue stream for regulatory agencies. However, such barriers are not insurmountable. While reliance can begin with a limited set of essential medicines, trust can be gradually built through joint inspections and regulator exchanges. Moreover, financing of the African Medicines Agency (AMA) can be made more predictable through member-state contributions rather than donor grants.

Another solution could be a reliance model in which agencies accept one another’s audits and those of the WHO. Firms may also continue to pay national fees, thus satisfying demands for harmonisation as well as the regulators’ financial self-sufficiency. The AMA and the African Continental Free Trade Area already provide frameworks for regulatory coordination and market integration [31]. Regionally, several initiatives are also in place to strengthen regulatory harmonisation and cooperation. The Indian Government may potentially be able to support these, for instance through the reciprocal stationing and training of regulatory officials and recognition arrangements for pharmacopeial standards.

### Escaping the local manufacturing trap

To achieve the continental ambition to meet at least 60% of Africa’s health product needs through local manufacturing by 2040 [33], modern and compliant drug manufacturing facilities will require multimillion-dollar investments. Moreover, trained quality-assurance and manufacturing personnel remain scarce, and economies of scale are undermined when medicine produced in one country cannot be sold in neighbouring markets without separate registration. Unreliable power and water, limited quality-control capacity and political instability add further costs and risks.

Although governments may require local manufacturing as a condition of market access, they often provide little or no procurement preference or trade protection, and they rarely waive additional audits for neighbouring markets. Two possible routes out of this trap warrant attention. The first is to sequence local production after regulatory consolidation, so that a plant in one African state can serve a regional market. The second route turns an apparent weakness into a potential platform. A majority of the world’s least developed countries (LDCs) are on the African continent. These countries benefit from transitional exemptions under the international intellectual property regime until 2033 [34]. This could allow the continent to become a site where Indian firms manufacture still-patented medicines for export to other LDCs. While these countries would gain technology and employment, India would gain access to an expanded market. Indian actors could also more proactively invest in joint ventures and production facilities on the continent, drawing on lessons of Indian companies that have already done so [35]. Such partnerships could potentially share costs and know-how, support training in good manufacturing practices, and be backed by long-term purchase commitments.

### From knowledge transfer to co-ownership

One of India’s most ambitious knowledge initiatives was the Pan-African e-Network. It linked Indian hospitals and universities with counterparts in around 35 African countries. The initiative appears to have been more successful in advancing tele-education than telemedicine [36]. The limited success of the telemedicine component was diagnostics. Clinicians in Malawi, Kenya and Ethiopia were confident in their own professional competence. They identified infrastructure, rather than knowledge, as the principal constraint.

The case illustrates a conceptual flaw in conventional approaches to ‘capacity building’. Capacity is often treated as something located in the recipient, rather than as a relational problem involving both parties. Indeed, learning typically runs in both directions. African achievements in disease control constitute an important reservoir of expertise. India’s own transitioning health system has much to learn from these experiences. One pharma industry veteran in Hyderabad summarised the approach required in these terms. India must be ‘a friend, not a teacher, and a partner, not the owner’.

## South–South interdependence and opportunity

In the near future, the India–Africa pharmaceutical relationship will most likely evolve through bilateral partnerships that are based on an understanding of win–win outcomes. Such arrangements cannot substitute for multilateral engagement because they are typically not free of asymmetries in power, capacity, and bargaining leverage. Nonetheless, Africa–India relations may offer a pragmatic basis for cooperation in a world where multilateralism is increasingly under pressure. Greater attention and resources should be directed to South–South interdependence and opportunity, rather than to restoring a global health architecture whose political and institutional moment may already have passed.

## Data Availability

Supporting data is not available, as participants did not consent for public sharing.
